# The Auditory-Brainstem Response to Continuous, Non-repetitive Speech Is Modulated by the Speech Envelope and Reflects Speech Processing

**DOI:** 10.3389/fncom.2016.00047

**Published:** 2016-05-26

**Authors:** Chagit S. Reichenbach, Chananel Braiman, Nicholas D. Schiff, A. J. Hudspeth, Tobias Reichenbach

**Affiliations:** ^1^Tri-Institutional Training Program in Computational Biology and Medicine, Weill Cornell Medical CollegeNew York, NY, USA; ^2^Howard Hughes Medical Institute and Laboratory of Sensory Neuroscience, The Rockefeller UniversityNew York, NY, USA; ^3^Department of Neuroscience, Brain and Mind Research Institute, Weill Cornell Medical CollegeNew York, NY, USA; ^4^Department of Bioengineering, Imperial College London, South Kensington CampusLondon, UK

**Keywords:** auditory brainstem response (ABR), speech processing, fundamental frequency, speech envelope, hearing

## Abstract

The auditory-brainstem response (ABR) to short and simple acoustical signals is an important clinical tool used to diagnose the integrity of the brainstem. The ABR is also employed to investigate the auditory brainstem in a multitude of tasks related to hearing, such as processing speech or selectively focusing on one speaker in a noisy environment. Such research measures the response of the brainstem to short speech signals such as vowels or words. Because the voltage signal of the ABR has a tiny amplitude, several hundred to a thousand repetitions of the acoustic signal are needed to obtain a reliable response. The large number of repetitions poses a challenge to assessing cognitive functions due to neural adaptation. Here we show that continuous, non-repetitive speech, lasting several minutes, may be employed to measure the ABR. Because the speech is not repeated during the experiment, the precise temporal form of the ABR cannot be determined. We show, however, that important structural features of the ABR can nevertheless be inferred. In particular, the brainstem responds at the fundamental frequency of the speech signal, and this response is modulated by the envelope of the voiced parts of speech. We accordingly introduce a novel measure that assesses the ABR as modulated by the speech envelope, at the fundamental frequency of speech and at the characteristic latency of the response. This measure has a high signal-to-noise ratio and can hence be employed effectively to measure the ABR to continuous speech. We use this novel measure to show that the ABR is weaker to intelligible speech than to unintelligible, time-reversed speech. The methods presented here can be employed for further research on speech processing in the auditory brainstem and can lead to the development of future clinical diagnosis of brainstem function.

## Introduction

The auditory-brainstem response (ABR) is an evoked potential generated from the auditory brainstem nuclei in response to auditory stimuli. Because the ABR can be measured noninvasively through scalp electrodes, it is widely used in both research and clinical settings to probe subcortical acoustic processing (Hood, 1998; Hall, 2007). Since the discovery of the ABR in 1970 by Jewett et al. (1970), a wealth of studies have investigated how the auditory brainstem processes a variety of acoustic signals. Such studies have mostly measured the ABR in response to simple stimuli such as clicks or pure tones. In particular, the auditory brainstem can exhibit a frequency-following response to the periodicity of a pure tone (Galbraith, 1994; Galbraith et al., 1995). The frequency-following response has a striking similarity to the eliciting periodic stimulus in both the temporal and the spectral domain. It presumably represents the phase-locked activity of neurons in the rostral brainstem, predominantly in the inferior colliculus, lateral lemniscus, and cochlear nucleus (Smith et al., 1975; Sohmer et al., 1977; Chandrasekaran and Kraus, 2010; Du et al., 2011).

Speech evokes a complex ABR that encodes many aspects of the complicated acoustic stimulus. A pioneering study in 1980 showed that formants are encoded in the speech-evoked ABR (Greenberg, 1980). Since then, a diverse set of speech stimuli, including Mandarin syllables, words such as “lily,” “apple,” and “piano,” consonant-vowel sounds, and short sentences have been used to elicit ABRs (Krishnan et al., 2004; Russo et al., 2004; Aiken and Picton, 2006; Parbery-Clark et al., 2009; Skoe and Kraus, 2010; Choi et al., 2013). Galbraith et al. (1995) demonstrated that the speech-evoked ABR resembles the eliciting stimulus so closely that it can be understood quite accurately by naïve participants when played to them as sound. It has further been demonstrated that the speech-evoked ABR can be affected significantly by aspects of the acoustic presentation, such as the level of environmental noise or whether the stimulation is monaural, dichotic, or diotic (Galbraith et al., 1998; Anderson and Kraus, 2010; Li and Jeng, 2011).

Important questions remain, however, regarding the role of the auditory brainstem in speech processing. Extensive efferent neural pathways project from higher areas of the auditory system such as the auditory cortex back to different areas of auditory brainstem, including the inferior colliculus and the cochlear nuclei. These connections suggest that the brainstem can play a role in high-level aspects of speech processing (Diamond et al., 1969; Weedman and Ryugo, 1996; Mulders and Robertson, 2000; Du et al., 2011; Barbas et al., 2013). Training in languages or in music can affect the subcortical processing of speech as measured through the speech-evoked ABR (Musacchia et al., 2007; Hornickel et al., 2012). Furthermore, short, repeated intelligible speech stimuli elicit a larger ABR than reversed, unintelligible speech (Galbraith et al., 2004). However, whether and how the ABR is modulated by higher cognitive functions such as attention and comprehension remains debated. Some researchers have measured a different latency of the peak response to click stimuli during attention to an auditory stimulus as opposed to other sounds (Brix, 1984; Ikeda et al., 2010), but other studies have found no significant difference (Collet and Duclaux, 1986; Connolly et al., 1989). Although the effect is small, the amplitude of the frequency-following response may be modulated by attention to pure tones (Galbraith and Doan, 1995; Galbraith et al., 2003). Attending to single vowels yields differences in the amplitude of the brainstem’s response, but the results are inconsistent between subjects (Lehmann and Schönwiesner, 2014). A reason for these dissonant findings may be the brevity of the signals employed, on the order of tens of milliseconds. Measuring the ABR requires several hundred to a thousand repetitions of the same stimuli, potentially allowing for neural adaptation and reducing the effect of efferent feedback (Lasky, 1997; Neupane et al., 2014).

Here we endeavored to measure the response of the auditory brainstem to continuous speech. To avoid potential adaptive affects to the stimulus we presented a non-repeating speech signal. We thus faced an important technical challenge: the ABR is of the order of microvolts and thus much smaller than the background electrical activity contributed by the cortex. Because we sought not to repeat the stimulus, we could not average the ABR over multiple repetitions and consequently could not establish the precise temporal waveform of the ABR. Instead we employed advanced data analytics to obtain meaningful features of the ABR from the recordings.

**Figure 1:**
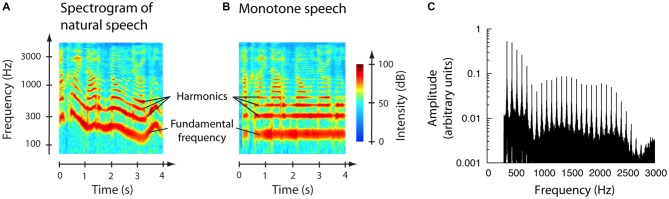
**Properties of natural and monotone speech. (A)** The spectrogram of a sample of natural speech shows that the energy concentrates at the fundamental frequency that typically lies between 100 Hz and 300 Hz and the corresponding harmonics. The fundamental frequency of speech and the harmonics vary in time. **(B)** Monotone speech has been modified to maintain constant frequencies of the fundamental and its harmonics. **(C)** The experiments employed monotone speech that was high-pass filtered at 300 Hz. The power spectrum reveals that the fundamental frequency, *f*_0_ = 89 Hz in this example, and its first two harmonics were absent from the speech sample.

Two important structural aspects of speech are promising in order to extract meaningful features of the ABR to continuous speech. First, every human voice has a distinct spectral structure: when a person speaks, the vocal folds open and close at a fundamental frequency that typically lies between 150 Hz and 250 Hz for a woman or between 100 Hz and 200 Hz for a man. Most of the spectrum of speech accordingly lies within distinct frequency bands, namely at the fundamental frequency and its more than 10 lowest harmonics (Figure 1A). The ABR to speech tracks this spectral structure: an amplitude spectrum of the response shows peaks at the fundamental frequency as well as its harmonics (Skoe and Kraus, 2010; Jeng et al., 2011). Although this response shows similarities to the frequency-following response to pure tones, a major difference exists. The speech-evoked ABR at the fundamental frequency is evoked not only by the fundamental frequency itself, but also by the harmonics in the speech signal. Indeed, the auditory brainstem exhibits a response at the fundamental frequency even when that frequency itself has been removed from the stimulus (Galbraith, 1994; Galbraith and Doan, 1995).

Second, the envelope of continuous speech traces important building blocks of speech, namely phonemes, syllables, and words. Cortical oscillations, especially in the delta and theta frequency bands, can entrain to the envelope of speech (Ding and Simon, 2012; Power et al., 2012; Horton et al., 2013; Peelle et al., 2013; Zion Golumbic et al., 2013). This neural entrainment is modulated by higher cognitive functions: it is stronger for an attended speech stream than for an unattended one (Horton et al., 2013) and may be larger for intelligible than for unintelligible speech (Peelle et al., 2013; Ding and Simon, 2014; Ding et al., 2014). The entrainment of cortical oscillations to the speech envelope may accordingly represent a neural mechanism for speech processing (Giraud and Poeppel, 2012).

Here we show that the fundamental frequency and the envelope of speech can be employed effectively to measure the ABR to continuous, non-repetitive speech. In particular, the brainstem responds to the fundamental frequency of a continuous, non-repetitive speech stream, the response is modulated by the envelope, and the envelope’s modulation greatly increases the signal-to-noise ratio of the ABR. Because continuous speech has a fundamental frequency that varies over time and thus hinders an assessment of the ABR (Figure 1A), we have used the computer-linguistic program Praat to convert natural speech into monotone speech in which the fundamental frequency and its higher harmonics remain constant throughout the speech stream (Figure 1B; Boersma, 2002; Deroche and Culling, 2011). This monotone speech is easily intelligible and complex enough to elicit sustained attention from human subjects. We then employ the developed method to investigate how the ABR to continuous speech is modulated by speech intelligibility.

## Materials and Methods

### Participants

Ten adult volunteers between 19 and 33 years of age participated in the experiments. All subjects had normal hearing and normal or corrected-to-normal vision and had no history of hearing or neurological impairments. All experimental methods were approved by the Imperial College Research Ethics Committee. All experiments were performed in accordance with relevant guidelines and regulations and every subject provided written informed consent prior to the experimental session.

### Monotone Speech Stimuli

Speech samples were obtained from publicly available audiobooks and were converted to monotone speech through the pitch-synchronous overlap-add (PSOLA) approach (Moulines and Charpentier, 1990) with the computer-linguistic software Praat (Boersma, 2002). The fundamental frequency of the speaker was set to 89 Hz. To prevent stimulus artifacts, every speech stimulus was high-pass filtered at three times the fundamental frequency of the speaker and thus did not contain the fundamental frequency and the first two harmonics. Reversed speech was created by temporally inverting a speech stimulus. Each speech stimulus lasted 3 min.

### Experimental Design

The experiment assessed whether the ABR provides information about speech processing by comparing responses to forward and to reversed speech. Each subject listened to both a forward and a reversed 3-min continuous speech stream. The order of the two speech streams was chosen randomly for every subject.

### Auditory-Brainstem Recordings

All recordings for the study were completed during a 3-week period. During each session subjects sat in a comfortable chair in a quiet room. Speech stimuli were presented to the subjects through custom electrically shielded earphones (hf5, Etymotic, USA) at a comfortable level of 70 dB SPL. Sound intensity was calibrated with a microphone (ECM8000, Behringer, Germany).

We measured responses from the auditory brainstem through active Ag/AgCl electrodes and a passive ground electrode (g.LADYbird and g.LADYbirdGND, Guger Technologies, Austria). The active electrodes were positioned at the cranial vertex (Cz) and on both mastoid processes. The passive ground electrode was placed on the central forehead (Lehmann and Schönwiesner, 2014). The impedance between each electrode and the scalp was measured (g.Zcheck, Guger Technologies, Austria) and confirmed to be below 5 kΩ. A bipolar amplifier (g.BSamp, Guger Technologies, Austria) enhanced the differences between the voltage signals at the mastoids and that at the vertex by a factor of 10,000 and band-pass filtered them between 0.1 Hz and 1 kHz. The analog voltage signals were digitized at a sampling frequency of 8 kHz with a data-acquisition card NI PCI 6221 (National Instruments, USA) and a custom-written Matlab program (MathWorks, USA). The Matlab program also presented speech signals to the subjects at a sampling frequency of 44.1 kHz through the computer’s internal sound card. The speech signals were time-locked to the electroencephalographic recordings and the voltage signals were saved for offline analysis.

### Analysis of Auditory-Brainstem-Response Signals

We first determined the significance of the brainstem’s response at the fundamental frequency by comparing it to the signal at neighboring frequencies. To quantify the latter signal, which constitutes the noise floor, we used Matlab to compute the average and the standard deviation of the Fourier amplitudes from 2 Hz below the fundamental frequency to 2 Hz above it, excluding the response at the fundamental frequency. We then considered the response at the fundamental frequency to be significant if its amplitude was at least three standard deviations above the mean response at the neighboring frequencies. We found that all responses were significant, and verified that the width of the frequency interval that was used to compute the noise floor did not impact this result.

We were then interested in the response of the auditory brainstem to continuous speech at the fundamental frequency, as well as in the modulation and correlation with the speech envelope. We thus employed four different methods to analyze the speech-evoked ABR. The methods were implemented using custom-written Matlab programs.

In the first method, we assessed the Fourier amplitude of the ABR at the fundamental frequency. The ABR is measured through surface electrodes that record a voltage signal *V*(*t*) from a starting time *t* = 0 to a final time *t_e_*. The analog signal is then sampled at a sampling frequency *F_s_* and thus transformed to a discrete signal ${\left\{V_{n}\right\}}_{n\;=0}^{N-1}$ with *N* = *t_e_F_s_*. The discrete Fourier transform decomposes the discrete time signal into its frequency components:

(1) $${\tilde{V}}_{k}\;=\;\sum_{n\;=\text{​ }0}^{N-1}V_{n}e^{-2\pi ikn/N},k\;=\;0,1,...,N-1$$

Because the temporal voltage signal is real, the complex Fourier coefficients $\tilde{V}$*_k_* fulfill the relation ${\tilde{V}}_{k}\;=\;{\tilde{V}}_{N-k}^{*}$ and the magnitude |$\tilde{V}$*_k_*| + |$\tilde{V}$*_N−k_*| = 2|$\tilde{V}$*_k_*| is the Fourier amplitude of the periodic component at frequency *f* = *k/t_e_*.

To determine the signal-to-noise ratio of the amplitude at the fundamental frequency, the voltage time series was divided into segments of 3 s duration. For each segment the Fourier component at the fundamental frequency was determined, and the responses from the left and the right brainstem were averaged. From the Fourier components of the segments we then computed the mean amplitude and standard deviation. The signal-to-noise ratio followed as the ratio of the mean amplitude to the standard deviation, that is, as the reciprocal of the coefficient of variation (Bushberg and Boone, 2011). We computed the signal-to-noise ratio for each subject’s ABR and from that obtained the population mean and its standard error for the signal-to-noise ratio.

As a second method, we determined the envelope-modulated ABR at the fundamental frequency. We first extracted the envelope of the speech signal *s*(*t*) through the Hilbert transformation,

(2) $$H\left[s\right](t)=\;-\frac{1}{\pi }\underset{{}_{\varepsilon \to 0}}{\lim}\int_{\varepsilon }^{\infty }\frac{s(t+\tau )-s(t-\tau )}{\tau }d\tau .$$

The speech envelope was obtained from the Hilbert transform by low-pass filtering at 30 Hz.

We then determined the voiced and voiceless components of the speech stream and their envelopes. The speech signal was divided into segments of 40 ms duration using Hann windows, and we computed the average speech envelope for each segment. We computed the power cepstrum for each segment,

(3) $$\text{power cepstrum}\;\text{=}\;{\left|{ℱ}^{-1}(log\left\{{\left|ℱ\left[s(t)\right]\right|}^{2}\right\})\right|}^{2},$$

in which ℱ denotes the Fourier transform (Benesty et al., 2008). We determined the amplitude at the quefrency that corresponded to the fundamental frequency of the speaker. If this amplitude was significantly higher than the average of the 10 neighboring quefrencies, or if the amplitude of the segment’s speech envelope exceeded a minimum threshold level, we considered that segment to correspond to the voiced part of speech, and otherwise to voiceless speech. For constructing the envelope of the voiced parts of speech, we kept the envelopes of all voiced segments but ignored those of voiceless segments. The envelope of the voiceless parts of speech was obtained analogously.

To compute the envelope-modulated ABR at the fundamental frequency, we downsampled the speech envelopes to the same sampling frequency as had been employed for the ABR. ${\left\{e_{n}\right\}}_{n\;=\;0}^{N-1}$ is the resulting discrete time series of the envelope for either the whole speech, its voiced parts, or its voiceless parts. Amplitude modulation of the ABR is then obtained by shifting the envelope by a temporal delay τ, and hence by an index *l = τF_s_* , with respect to the ABR and by multiplying both signals. We consider the envelope before the speech starts, that is, before time *t* = 0, to be zero. The envelope-modulated ABR at the fundamental frequency, which we denote by $\tilde{V}$^(env.mod.)^(*τ*), follows from the Fourier amplitude at the fundamental frequency:

(4) $${\tilde{V}}_{\text{k}}^{\left(\text{env}.\text{mod}.\right)}(\tau )\;=\;\sum_{n\;=\;0}^{N-1}V_{n}e_{n-l}e^{-2\pi ikn/N}.$$

The index *k* is chosen such that it corresponds to the fundamental frequency *f_0_*, that is, *f_0_* = *k/t_e_*.

We computed the envelope-modulated ABR at the fundamental frequency by first dividing the ABR signal and the corresponding speech envelope into 3 s segments. We then computed the envelope-modulated ABR at the fundamental frequency for each segment and for temporal delays from τ = −300 to τ = 500 ms, and found a peak at the characteristic delay of about τ = 10 ms. For every segment we thus computed the peak amplitude as the mean of the envelope-modulated ABR at delays between τ = 0 and τ = 20 ms. The envelope-modulated ABR at the fundamental frequency and at the characteristic latency for an individual recording followed as the mean of these peak amplitudes across all segments; the standard deviation across the different segments yielded the variation in the envelope-modulated ABR. The signal-to-noise ratio followed as the ratio of the mean to the standard deviation. We computed this ratio for each subject individually, and then used the obtained data to determine the population mean and its standard error.

As a third method, we employed a short-time Fourier transformation to extract the timecourse of the ABR at the fundamental frequency, and then determined the correlation of this timecourse with the speech envelope. For short-time Fourier transformation, we partitioned the voltage time series into segments of 80 ms duration through Hann windows and computed the Fourier transform for each segment. For every segment we extracted the Fourier amplitude at the fundamental frequency and thus found the discrete timecourse of the ABR at that frequency. We denote this discrete time series, with the mean subtracted and consisting of a number of *M* of data points, by ${\left\{A_{n}\right\}}_{n\;=0}^{M-1}$. We downsampled the speech envelope to have the same sampling frequency *F*_ABR_ as the timecourse of the ABR at the fundamental frequency, and subtracted the mean, resulting in the discrete envelope time series ${\left\{E_{n}\right\}}_{n\;=\;0}^{M-1}$. We computed the cross-correlation of the speech envelope, shifted by various delays corresponding to an index *m = τF_ABR_*, with the discrete timecourse of the ABR at the fundamental frequency:

(5) $$(S*E)(\tau )\;=\;\frac{1}{M\sigma_{A}\sigma_{E}}\sum_{n\;=\;0}^{M-1}A_{n}E_{n-m}$$

in which σ_A_ denotes the standard deviation of the ABR timecourse ${\left\{A_{n}\right\}}_{n\;=0}^{M-1}$, and *σ_E_* denotes the standard deviation of the envelope ${\left\{E_{n}\right\}}_{n\;=\;0}^{M-1}$. As for the envelope-modulated signal, the correlation exhibited a peak at a delay of *τ* = 10 ms. We extracted the signal-to-noise ratio around the peak from the temporal correlation from τ = 0 to τ = 20 ms analogously to the signal-to-noise ratio of the envelope-modulated ABR.

As a fourth method, we used a wavelet transformation to extract the timecourse of the ABR at the fundamental frequency. To this end we employed a morlet wavelet as the mother wavelet at a temporal standard deviation of 16 ms and at the fundamental frequency. The morlet wavelet was chosen to capture the oscillatory behavior of the ABR. We then correlated the obtained timecourse of the ABR to the speech envelope as in the third method.

### Statistical Analysis

To determine the statistical significance of the differences in the signal-to-noise ratios between the four different methods for extracting the ABR response—the simple Fourier transformation, the cross-correlation of the speech envelope with the timecourse of the ABR at the fundamental frequency determined either through a short-time Fourier transformation or through a wavelet transformation, and the envelope-modulated ABR at the fundamental frequency—we performed two-sample Student’s *t*-tests for pairwise comparisons of the signal-to-noise ratios obtained by these four methods. Although we employed the Bonferroni correction to account for the six pairwise comparisons, the level of statistical significance for each statistical test did not depend on whether this correction was used.

To assess differences in the ABR to forward and to the time-reversed speech on the population level, we computed the envelope-modulated ABR for forward and reverse speech, for each subject and at a range of delays. We then computed the mean and standard error of the mean of these responses across all individuals. To investigate statistical significance, we analyzed the responses around the peak latency of 10 ms by averaging the responses at latencies between 0 and 20 ms for each individual subject, and from that computed the mean and standard error of the mean across all subjects. We performed a two-sample Student’s *t*-test to assess whether the difference in the mean amplitudes for the forward and the time-reversed conditions was statistically significant.

To investigate differences in the ABR to forward and reverse speech on the level of individual subjects, we computed the envelope-modulated ABR at the fundamental frequency and at the characteristic latency as described above. We obtained the mean and the standard error of the mean across all segments. This gave us an average response as well as a measure of the variability of the envelope-modulated ABR in an individual subject. To assess the statistical significance of the difference in the responses to forward and to reverse speech in an individual subject, we performed a paired, two-sample Student’s *t*-test.

The results of the statistical tests are indicated in the figures through asterisks: no asterisk is given when results are not significant (*p* > 0.05), one asterisk when results are significant (*0.01 < *p* < 0.05), two asterisks when significance is high (**0.001 < *p* < 0.01), and three asterisks when significance is very high (***p < 0.001).

## Results

**Figure 2:**
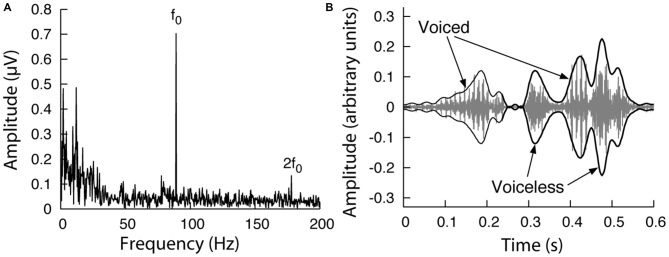
**Response of the auditory brainstem to continuous monotone speech. (A)** The power spectrum of the auditory-brainstem response (ABR) to 3 min of monotone speech shows a strong response at the fundamental frequency (*f_0_*) and at its second harmonic (2*f*_0_). **(B)** A speech waveform (gray) is characterized by variations on fast and slow time-scales. Slow variations, on the order of 100 ms and above, define the speech envelope (black) that traces distinct syllables and words. Voiced parts of speech are characterized by a periodicity at the fundamental frequency; voiceless parts lack this periodic structure.

We recorded ABRs from healthy volunteers presented with a monotone speech stream. To avoid stimulus artifacts we removed the fundamental frequency and the first two harmonics from the speech (Figure 1C), thus ensuring that any signal measured by the electrodes at those frequencies did not result from the electrical activity of the earphones. We found that the brainstem exhibited a reliable response at the constant fundamental frequency and at higher harmonics of the monotone speech (Figure 2A). Because it was the largest and most informative regarding speech processing, we focused on the amplitude at the fundamental frequency. The absence of a response at the fundamental frequency in control recordings, in which the earphones were near the ear but not in the ear canal so that subjects could not hear the speech, confirmed that the measured ABR at the fundamental frequency was not a stimulus artifact.

### Modulation of the ABR with the Speech Envelope

Speech includes voiced and voiceless components (Figure 2B). The voiceless part contains a broad range of frequencies. The voiced elements result from vowels, among other features, and exhibit a distinct spectral structure with a fundamental frequency and many harmonics. Because the ABR to speech at the fundamental frequency arises from the voiced parts of the speech, we hypothesized that the timecourse of the ABR at the fundamental frequency is modulated by the envelope of the voiced parts of speech.

To investigate the envelope modulation of the ABR, we multiplied the measured brainstem response by the envelope of the voiced components of the speech at different temporal delays. The amplitude of the resulting signal at the fundamental frequency was then determined through spectral analysis at each temporal delay; we refer to this signal as the *envelope-modulated ABR at the fundamental frequency*. If the ABR at the fundamental frequency results from the voiced parts of speech, then the envelope-modulated ABR at that frequency should have a peak at a characteristic delay that corresponds to the latency between the speech stimulus and the neural response in the brainstem. We also computed the modulation of the ABR by the envelope of the entire speech and by the envelope of the voiceless parts. The modulation of the ABR by the envelope of the whole speech signal should yield a peak at the same latency, albeit with a smaller magnitude. Modulating the ABR by the envelope of the voiceless components alone should not produce a peak, for these speech components do not yield an ABR at the fundamental frequency.

**Figure 3:**
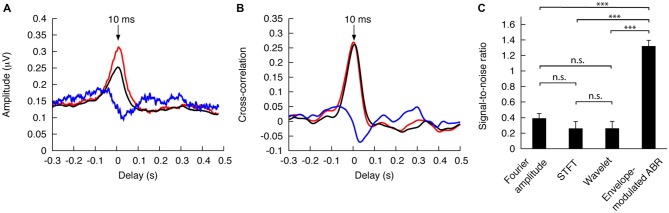
**Envelope-modulated ABR and cross-correlation of the ABR timecourse to the speech envelope. (A)** Modulation of the ABR with the envelope of the voiced parts of speech (red) as well as with the envelope of the whole speech signal (black) yields a peak at a delay of 10 ms. Envelope modulation of the ABR with the voiceless parts of speech, however, produces a minimum value at a delay of about 35 ms. **(B)** The cross-correlation of the timecourse of the ABR at the fundamental frequency with the voiced parts of speech (red) and the envelope of the entire speech stimulus (black) exhibit likewise a maximum at the delay of 10 ms, whereas the cross-correlation with the envelope of the voiceless speech components yields a minimum at a delay of 35 ms. **(C)** The signal-to-noise ratio of the envelope-modulated ABR at the fundamental frequency, and at the delay of 10 ms, is several fold larger than that obtained by simple Fourier transformation. It also significantly exceeds the cross-correlation of the speech envelope with the timecourse of the ABR, at the delay of 10 ms, both when the ABR timecourse is extracted through short-time Fourier transfomation (STFT) and when it is identified by a wavelet transform (*** *p* < 0.001; n.s., not significant).

For modulation by the envelope of the entire speech or by the envelope of the voiced parts we measured a peak in the envelope-modulated ABR at the fundamental frequency at a delay of 10 ms (Figure 3A). The peak was larger for the correlation with the voiced components of the speech than for that with the whole speech. Modulation by the voiceless parts of the speech yielded a negative peak at around 35 ms. These results indicate that the brainstem response to the fundamental frequency reflects primarily the voiced components of the speech. The characteristic latency of the response exceeds those of the peaks in the standard click-evoked ABR, but corresponds to the latency observed in the ABR to vowels (Skoe and Kraus, 2010).

### Improving the Signal-to-Noise Ratio Through Modulating or Correlating the ABR with the Speech Envelope

Modulating the ABR with the voiced speech envelope at the characteristic delay of 10 ms largely eliminates the periods in a recording during which the brainstem does not respond at the fundamental frequency. We thus expected that the modulation of the ABR with the speech envelope would reduce the noise in the recordings. To quantify the putative noise reduction, we computed the signal-to-noise ratio for the amplitude of the peak in the envelope-modulated ABR at the fundamental frequency. To obtain an estimate of the inter-subject variability of the signal-to-noise ratio, we calculated the ratio for each individual and determined the population mean and its standard error. We also calculated the signal-to-noise ratio for the Fourier amplitude of the ABR at the fundamental frequency, without modulating the signal by the speech envelope, and determined the population mean and the associated standard error. We found that, by taking the envelope modulation of the ABR, we obtained a signal-to-noise ratio of the amplitude at the fundamental frequency that was more than threefold as large as when we computed the Fourier amplitude of the ABR alone (Figure 3C). The difference was highly significant (*p* < 0.001).

Another method to determine how the voiced parts of speech shape the ABR at the fundamental frequency is to investigate the cross-correlation between the timecourse of the ABR and the envelope of the voiced parts of speech. This correlation might also improve the signal-to-noise ratio. We computed the timecourse of the ABR at the fundamental frequency by dividing the time series into short time windows and analyzing the Fourier amplitude at the fundamental frequency in each window (short-time Fourier transformation). As another method, we computed the timecourse of the ABR at the fundamental frequency through a wavelet transform using the morlet wavelet as the mother wavelet. Each of these two resulting timecourses was then correlated with the envelope of the voiced parts of speech. As for the envelope-modulated ABR at the fundamental frequency, the cross-correlation exhibited a peak at a delay of 10 ms (Figure 3B). We then computed the signal-to-noise ratio of the correlation around the characteristic latency for each subject, and calculated the corresponding population mean and its standard error. We found that the differences in the signal-to-noise ratios that were obtained from the Fourier amplitude as well as from the correlation values as computed from the short-time Fourier transformation and the wavelet transformation were all statistically insignificant (*p* > 0.05). The signal-to-noise ratio of the envelope-modulated ABR at the fundamental frequency and at the characteristic delay was, however, severalfold larger than the signal-to-noise ratios obtained using the other methods, and the differences were highly significant (*p* < 0.001; Figure 3C). The best signal-to-noise ratio thus resulted not from cross-correlating the timecourse of the ABR with the speech envelope, but rather from modulating the ABR by the speech envelope at the characteristic delay and then extracting the amplitude at the fundamental frequency.

Motivated by the substantial increase in the signal-to-noise ratio of the envelope-modulated ABR at the fundamental frequency as opposed to a simple Fourier transform of the ABR, we employed this measure—the envelope-modulated ABR at the fundamental frequency—to investigate how the ABR to continuous speech is modulated by speech intelligibility.

### Modulation of the ABR by Speech Intelligibility

We investigated the influence of the intelligibility of continuous, non-repetitive speech on brainstem activity by presenting subjects with forward and time-reversed monotone speech. Although the two stimuli have an identical spectral composition, only the forward speech is intelligible. Comparison of the neural responses to the two stimuli has previously been used to investigate speech comprehension, to diagnose brain function, and to identify the role of the auditory brainstem in speech processing (Schiff et al., 2005; Deng and Srinivasan, 2010; Howard and Poeppel, 2010; Sunami et al., 2013).

**Figure 4:**
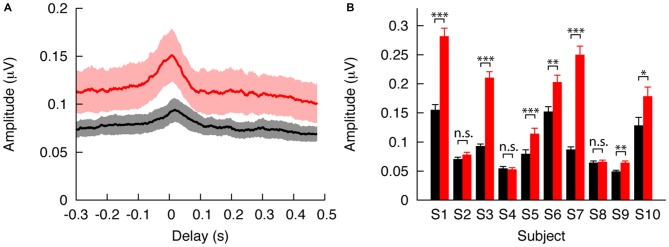
**Modulation of the ABR by speech intelligibility. (A)** The envelope-modulated ABR to unintelligible reverse speech (red) exceeds that to intelligible forward speech (black) when averaged over all subjects. The envelope-modulated ABR to both speech stimuli is largest around the characteristic delay of 10 ms. The standard errors of the mean (shaded) that follow from the variability between the subjects are smaller than the difference between the mean responses, and this difference is statistically significant. **(B)** For every subject, the response to time-reversed monotone speech (red) exceeds the neural response to forward monotone speech (black). The difference is statistically significant in the majority of the study participants (* 0.01 < *p* < 0.05; ** 0.001 < *p* < 0.01; *** *p* < 0.001; n.s., not significant).

We computed the envelope-modulated ABR at the fundamental frequency for each subject, and from that the population mean and its standard error (Figure 4A). We found that the envelope-modulated ABR to reversed speech exceeded that to forward speech by about half for every latency. By computing the mean amplitude at the peak of the envelope-modulated ABR, we found that the difference was statistically significant (*p* < 0.05).

We then investigated whether the responses to forward and to reverse speech also differed significantly and consistently at the level of individual subjects. For each individual we computed the envelope-modulated ABR to forward and time-reversed speech at the fundamental frequency and at the characteristic latency. Nine out of 10 subjects had a larger envelope-modulated ABR at the fundamental frequency for reverse than for forward speech (Figure 4B). These differences were statistically significant in seven of the nine subjects (*p* < 0.05). Only one subject showed a larger response to forward than to reverse speech, but the difference was insignificant (*p* > 0.5).

## Discussion

Our results demonstrate that important structural features of the brainstem’s response to continuous speech can be detected reliably by electrophysiological means. Although we cannot measure the precise temporal form of the ABR, as is feasible through repetitive measurements with short acoustic stimuli such as clicks or vowels, we can extract and quantify structural features of the ABR that emerge in response to characteristics of continuous speech. We have found specifically that the brainstem responds at the fundamental frequency of monotone speech even when that frequency is absent from the speech stimulus. Our results additionally demonstrate that the ABR at the fundamental frequency is modulated by the envelope of the voiced part of speech and that the timecourse of the ABR is correlated to the envelope.

The envelope modulation of the ABR can be employed to reduce significantly the noise in the response at the fundamental frequency. Whereas a Fourier transform of a 3-min recording of the ABR to continuous speech yields a signal-to-noise ratio of only 0.4 for the amplitude at the fundamental frequency, modulation of the ABR by the envelope of the voiced parts of speech, at the characteristic delay and at the fundamental frequency, achieves a signal-to-noise ratio of 1.3. Because the response of the auditory brainstem at the fundamental frequency results from the voiced parts of speech alone, focusing on those components reduces the noise in a recording. Although we likewise expect the correlation of the timecourse of the ABR to the speech envelope to reduce the noise, the resulting signal-to-noise ratio is below that obtained when modulating the ABR with the envelope, and even below the signal-to-noise ratio of the Fourier amplitude. This deficiency likely stems from the short-time Fourier transformation that is required to extract the timecourse of the ABR, which can then be correlated to the envelope. Short-time Fourier transformation has a poor frequency resolution that varies inversely to the duration of the time window. The poor frequency resolution renders the timecourse of the ABR at the fundamental frequency much noisier than the signal obtained by a Fourier transform over a longer recording, as we can employ for the envelope-modulated ABR at the fundamental frequency. The same reasoning applies to the wavelet transform and can explain why this method also yields a small signal-to-noise ratio.

The increase of the signal-to-noise ratio by more than a factor of three through modulation of the brainstem response by the envelope of the voiced parts of speech can accelerate auditory-brainstem recordings significantly. According to the central limit theorem, a longer recording improves the signal-to-noise ratio in proportion to the square root of the duration of the recording. Raising the signal-to-noise ratio by a factor of 3 therefore requires a ninefold longer recording. Conversely, because the method proposed here increases the signal-to-noise ratio by more than a factor of 3 through computational means, we can reduce the recording time by more than ninefold and still obtain a signal-to-noise ratio similar to that for the longer recording with a simple Fourier analysis. Although the additional numerical analysis requires several layers of computation, all of them can run in real time.

Our results on the influence of speech intelligibility on the ABR differ from previous findings. An earlier study addressed differences in the ABR to short, repetitive speech signals and their time-reversed versions and found that forward speech elicited a stronger response at the fundamental frequency than did reverse speech (Galbraith et al., 2004). We have observed the opposite result in response to continuous non-repetitive speech: forward, intelligible speech yields a smaller ABR at the fundamental frequency than does reverse, unintelligible speech. The discrepancy between the two studies may reflect differences in how the brain responds to many repetitions of the same short speech signal rather than to a long, non-repetitive, continuous stream of speech. In particular, the brain may adapt to many repeated presentations of the same speech stimulus, an effect that can be avoided through the non-repetitive speech signal that we have employed here.

Although forward and reverse speech have the same Fourier spectrum, the two signals differ phonetically. The manner in which voiceless consonants transition into voiced components can differ between the stimuli; owing to nonlinear processing by the cochlea, this can lead to differences in the brainstem response (Dau, 2003). However, in our study we have shown that the ABR at the fundamental frequency results from the voiced parts of the monotone speech, components that we expect to be comparable between forward and reverse speech. Further studies are needed to clarify whether the differences in phonetical structure between forward and reverse monotone speech, associated with the transition of consonants to vowels, cause a difference in the brainstem’s response.

In this study we have focused on the response at the fundamental frequency only. However, the brainstem also responds at higher harmonics, and these neural signals likely contain information about speech processing as well. Moreover, empirical mode decomposition such as through the Hilbert-Huang transform can extract nonlinear oscillations from a time series, which may be employed to identify nonlinear responses at the fundamental frequency as well as at higher harmonics (Huang and Shen, 2005). Investigating these issues will further clarify the role of the auditory brainstem in speech processing.

ABRs are used routinely to evaluate hearing, specifically to assess the integrity of the ear and the brainstem. The results presented here suggest that the ABR to continuous monotone speech can provide valuable information both about the integrity of the brainstem and about auditory processing. Moreover, forward vs. time-reversed speech stimuli have been used clinically to assess patients in a minimally conscious state (Schiff et al., 2005). Measuring the envelope-modulated ABR to forward and to reverse speech may likewise provide a valuable tool in assessing auditory processing in patients suffering from disorders of consciousness (Giacino et al., 2002; Laureys et al., 2004; Schiff, 2010; Goldfine et al., 2011).

## Author Contributions

CSR, CB, NDS, AJH and TR designed the research, performed the experiments, analyzed the data, and prepared the manuscript.

## Conflict of Interest Statement

The authors declare that the research was conducted in the absence of any commercial or financial relationships that could be construed as a potential conflict of interest.
